# Evidence for Altered Epigenetic Regulatory Machinery in Migraine: A Peripheral Blood Study

**DOI:** 10.3390/ijms27167182

**Published:** 2026-08-11

**Authors:** Michal Fila, Kinga Kołacz-Milewska, Małgorzata Grygiel, Lukasz Przysło, Karolina Tworek, Agnieszka Robaszkiewicz, Maksim Ionov, Janusz Blasiak

**Affiliations:** 1 Department of Developmental Neurology and Epileptology, Polish Mother’s Memorial Hospital Research Institute, 93-338 Lodz, Poland; lukasz.przyslo@iczmp.edu.pl; 2 Laboratory of Microscopic Imaging and Specialized Biological Techniques, University of Lodz, 90-237 Lodz, Poland; kinga.kolacz-milewska@biol.uni.lodz.pl; 3 The Bio-Med-Chem Doctoral School of the University of Lodz and Lodz Institutes of the Polish Academy of Sciences, 90-237 Lodz, Poland; 4 Department of General Biophysics, University of Lodz, 90-237 Lodz, Poland; malgorzata.grygiel@biol.uni.lodz.pl (M.G.); agnieszka.robaszkiewicz@biol.uni.lodz.pl (A.R.); maksim.ionov@biol.uni.lodz.pl (M.I.); 5 Faculty of Medicine and Health Sciences, Calisia University, 62-800 Kalisz, Poland; k.tworek@uniwersytetkaliski.edu.pl; 6 Faculty of Health Sciences, Collegium Medicum, Mazovian University in Plock, 09-402 Plock, Poland; 7 Faculty of Medicine, Collegium Medicum, Mazovian University in Plock, 09-402 Plock, Poland

**Keywords:** migraine, epigenetics, CGRP, TRPA1, HDAC1, HDAC6, systemic alterations in migraine

## Abstract

Calcitonin gene-related peptide (CGRP), encoded by the *CALCA* gene, plays a central role in migraine pathophysiology; however, it remains unclear whether migraine is associated with persistent systemic molecular alterations affecting the CGRP pathway during the interictal phase. Transient receptor potential cation channel subfamily A member 1 (TRPA1) stimulates the release of CGRP from nerve endings. We investigated gene-specific and global epigenetic regulatory mechanisms associated with *CALCA* and *TRPA1* expression in the peripheral blood of 56 patients with episodic migraine in the interictal phase and 36 headache-free controls. Expression of *CALCA*, *TRPA1*, and genes encoding epigenetic regulators, histone deacetylases 1 and 6 (HDAC1 and HDAC6), was assessed by quantitative real-time PCR. DNA methylation of *CALCA* and *TRPA1* was evaluated using methylation-specific PCR. Expression of other epigenetic regulators miRNA-375, miRNA-382, and miRNA-34a was determined using TaqMan MicroRNA assays. No significant differences were observed between migraine patients and controls in *CALCA* or *TRPA1* expression, nor in DNA methylation of these genes. Similarly, no differences were found in the expression of miRNA-375 and miRNA-382. In contrast, miRNA-34a expression was significantly reduced in migraine patients. Moreover, the expression of *HDAC1* and *HDAC6* was significantly decreased in migraine. Despite the established role of CGRP in migraine, no evidence of persistent systemic dysregulation of *CALCA* or *TRPA1* was detected in peripheral blood during the interictal phase. However, altered expression of miRNA-34a, *HDAC1*, and *HDAC6* suggests the presence of broader epigenetic and post-transcriptional regulatory disturbances. These findings support a model in which migraine is associated with dysregulation of regulatory mechanisms rather than stable alterations of individual target genes.

## 1. Introduction

Migraine is a chronic, common and disabling neurological disorder characterized by recurrent, usually unilateral, headache attacks, often accompanied by sensory disturbances [1]. Despite its high prevalence, the molecular mechanisms underlying migraine remain incompletely understood. One of the main reasons for this is the limited accessibility of biologically relevant target tissues. However, migraine is not limited to headaches, and the current view is that it is a systemic neurologic disorder that can affect multiple body systems before, during, and after the headache phase [2]. In a clinical context, symptoms such as fatigue, yawning, nausea, neck stiffness, food cravings, and increased urination are often considered the most characteristic systemic manifestations of migraine [3]. However, the knowledge about systemic molecular signs of migraine is scarce, and there are no validated molecular “signs” of migraine used routinely in clinical practice. Migraine remains a clinical diagnosis based largely on patient-reported symptoms and may therefore be influenced by subjective perception and recall bias [4]. As the target tissue is inaccessible, great interest is focused on systemic migraine signs. Several studies report systemic molecular changes, and candidate biomarkers have been identified in blood, saliva, cerebrospinal fluid, and other biofluids [5].

Calcitonin gene-related peptide (CGRP), encoded by the *CALCA* gene, is considered a key mediator in migraine pathophysiology [6]. Experimental studies have shown that administration of CGRP can induce migraine-like attacks, while CGRP-targeted therapies have demonstrated clinical efficacy [7]. Transient receptor potential cation channel subfamily A member 1 (TRPA1) is an ion channel that can be activated by oxidative stress products, leading to the release of CGRP from nerve endings [8]. TRPA1 may represent a key component of the signaling pathway linking oxidative stress and nitric oxide (NO) production to CGRP release, thereby potentially contributing to migraine initiation [9]. In addition, TRPA1 has been implicated in nociceptive signaling and neurogenic inflammation, suggesting its potential role in migraine-related mechanisms [10].

However, most evidence regarding CGRP and TRPA1 derives from studies focusing on the central nervous system or acute experimental conditions. It remains unclear whether these mechanisms are associated with systemic molecular alterations, particularly during the interictal phase.

Epigenetic mechanisms, including DNA methylation, microRNA-mediated regulation, and histone covalent post-translational modifications, have increasingly been recognized as important modulators of gene expression in neurological disorders [11]. Exploring epigenetic aspects of migraine pathogenesis may provide insight into whether migraine is associated with persistent changes in gene regulation or reflects a more dynamic and context-dependent process.

We have previously proposed that epigenetic regulation of the *CALCA* gene may represent a promising target for improving the efficacy and safety of migraine treatment [12]. Both CGRP and TRPA1 may be important for the effects of oxidative stress, mediated by reactive oxygen and nitrogen species (RONS) in migraine [13]. Recently, we suggested that epigenetic modifications affecting the RONS–TRPA1–CGRP axis may play a significant role in the pathogenesis of migraine and other pain-related syndromes [14].

In this study, we evaluated the expression of the *CALCA* and *TRPA1* genes in the peripheral blood of migraine patients and headache-free controls. We also measured DNA methylation levels in the promoters of these genes. Moreover, we investigated the expression of several miRNAs that are important for the regulation of *CALCA* and/or *TRPA1*, including miR-375, miR-382, and miR-34a. Because epigenetic regulation operates at both gene-specific and global levels, we also investigated the expression of the histone deacetylase 1 (*HDAC1*) and 6 (*HDAC6*) genes. Histone deacetylases encoded by these genes are key components of the epigenetic regulatory machinery involved in chromatin remodeling and transcriptional regulation and may reflect broader alterations in cellular regulatory networks that are not detectable by analysis of individual target genes alone [15]. Thus, the present study addressed both local regulatory mechanisms directly affecting *CALCA* and *TRPA1* expression (DNA methylation and miRNA-mediated regulation) and broader epigenetic regulatory pathways represented by *HDAC1* and *HDAC6* expression.

## 2. Results

### 2.1. Patients

The study included 56 patients with episodic migraine and 36 headache-free controls. Clinical and demographic characteristics of the migraine cohort are presented in Table 1.

The study group was predominantly female and consisted exclusively of patients with episodic migraine. Overall, the cohort was characterized by a moderate-to-high disease burden, as reflected in headache frequency and migraine-related questionnaire scores.

### 2.2. CALCA and TRPA1 Expression

**Figure 1:**
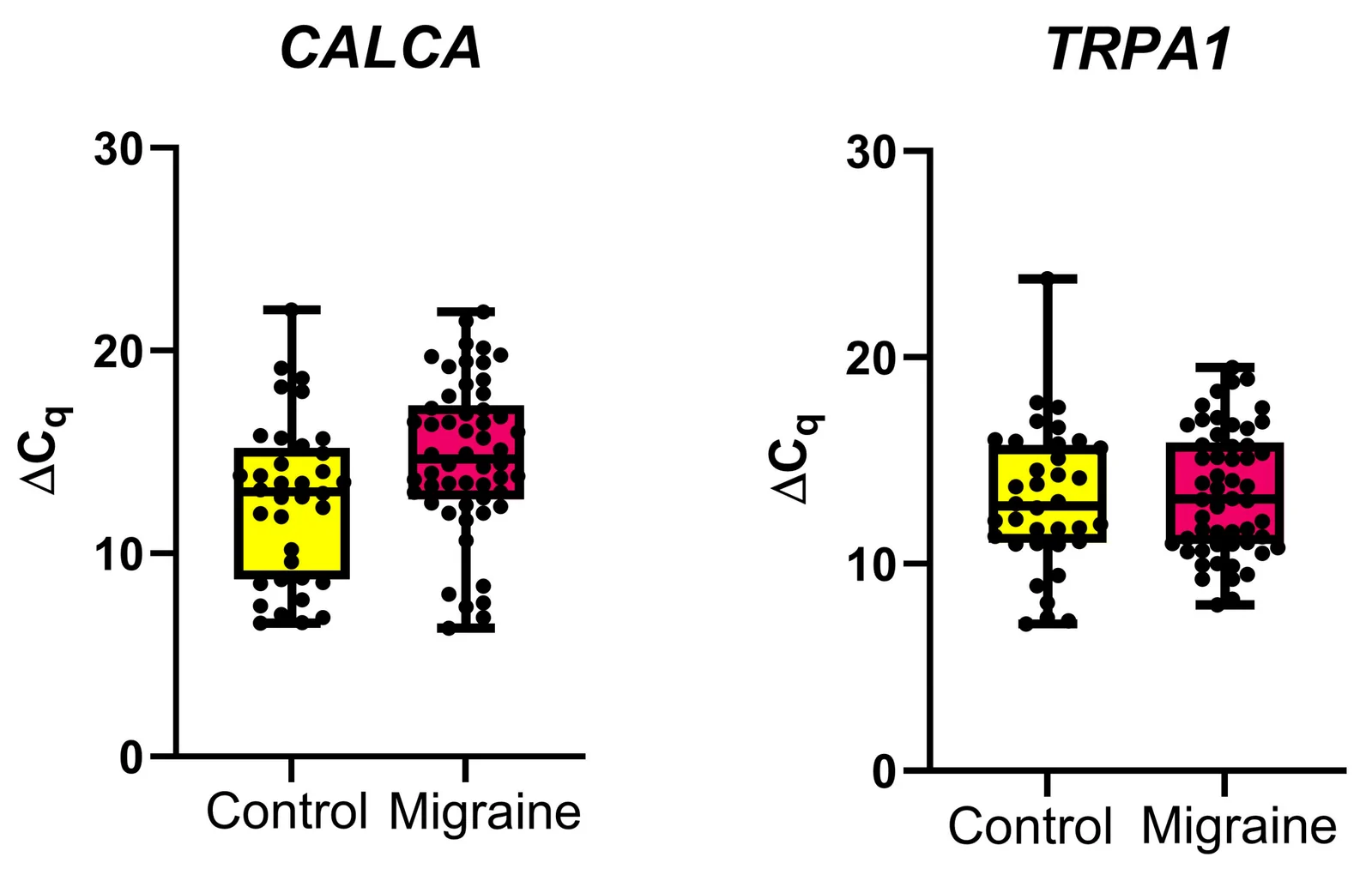
Expression of the calcitonin-related polypeptide alpha (*CALCA*) and transient receptor potential ankyrin 1 (*TRPA1*) genes in migraine patients (n = 54) and controls (n = 32). Gene expression was assessed by quantitative real-time PCR and expressed as ΔCq values (Cq_target − Cq_reference), with lower values indicating higher expression levels. Data are presented as box-and-whisker plots with individual data points. The central line represents the median, boxes indicate the interquartile range (IQR), and whiskers denote the minimum to maximum values.

The expression level of the *CALCA* and *TRPA1* genes is presented in Figure 1.

For *CALCA* expression, no significant differences were observed between migraine and control groups. Although minor differences in median values were noted, the distributions of ΔCq values showed substantial overlap between groups. Similarly, *TRPA1* expression did not differ significantly between migraine and control groups. The distributions of ΔCq values were comparable, with pronounced overlap and similar medians. Overall, the expression of both *CALCA* and *TRPA1* showed considerable inter-individual variability, with no evidence of group-specific differences.

### 2.3. DNA Methylation of the CALCA and TRPA1 Genes

**Figure 2:**
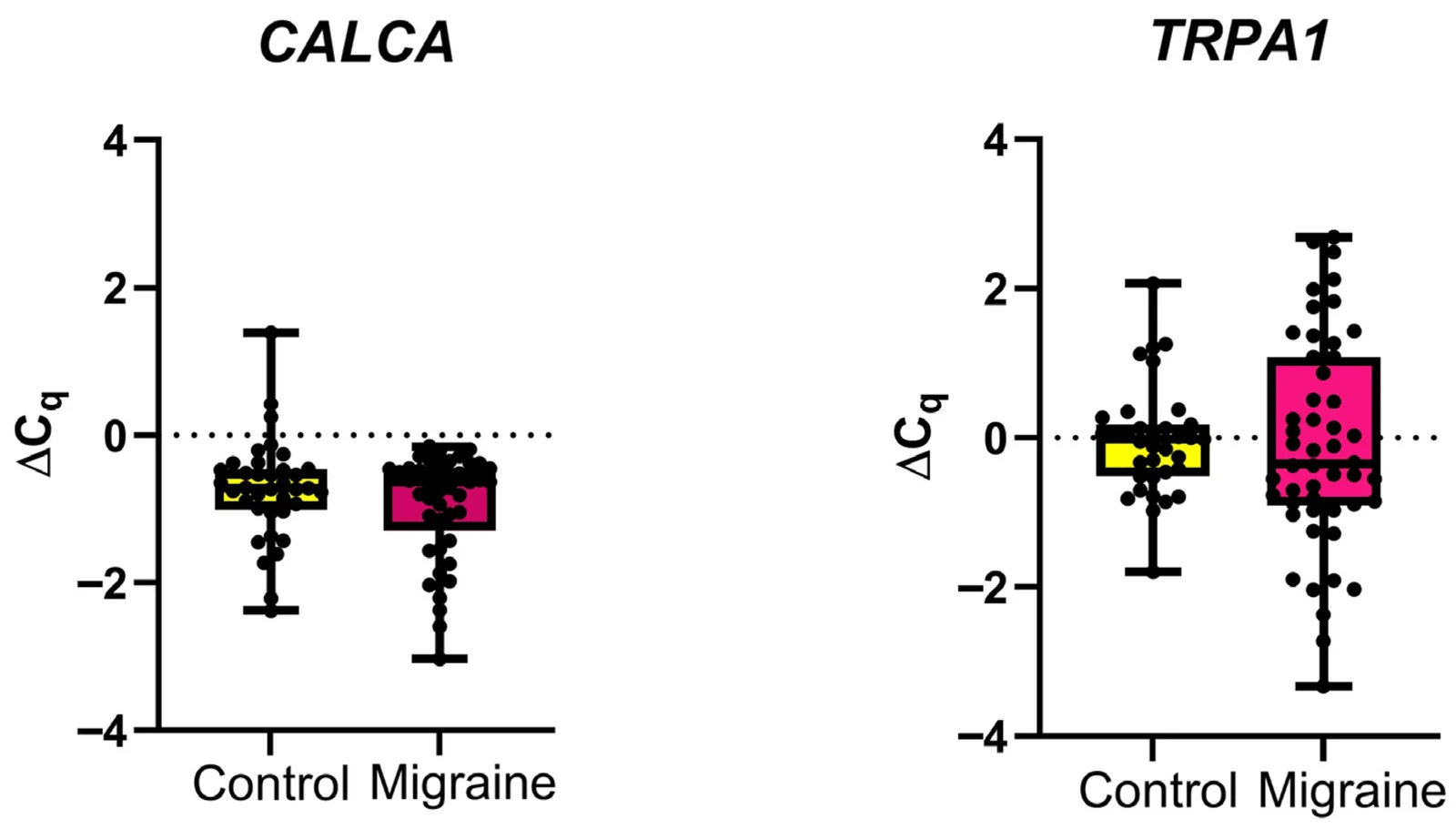
DNA methylation levels of the calcitonin-related polypeptide alpha (CALCA) and transient receptor potential ankyrin 1 (TRPA1) genes in migraine patients (n = 45 and 50, respectively) and controls (n = 37 and 35, respectively). DNA methylation was assessed using methylation-specific PCR and expressed as a difference between Cq values for methylated (M) and unmethylated (U) DNA—ΔCq = Cq_M − Cq_U, where lower values correspond to a higher proportion of methylated DNA. Data are presented as box-and-whisker plots with individual data points. The central line represents the median, boxes indicate the interquartile range (IQR), and whiskers denote the minimum to maximum values.

DNA methylation levels of the CALCA and TRPA1 genes are presented in Figure 2.

No statistically significant differences in ΔCq values were observed between migraine and control groups for either gene. For *CALCA*, a slight shift toward lower ΔCq values was observed in the migraine group; however, the distributions showed substantial overlap between groups. Similarly, *TRPA1* methylation did not differ between groups, with comparable distributions and a pronounced overlap of ΔCq values. Notably, a broader range of values was observed in the migraine group, indicating increased inter-individual variability. Overall, DNA methylation patterns of both *CALCA* and *TRPA1* were similar in migraine patients and controls.

### 2.4. miRNA Expression

**Figure 3:**
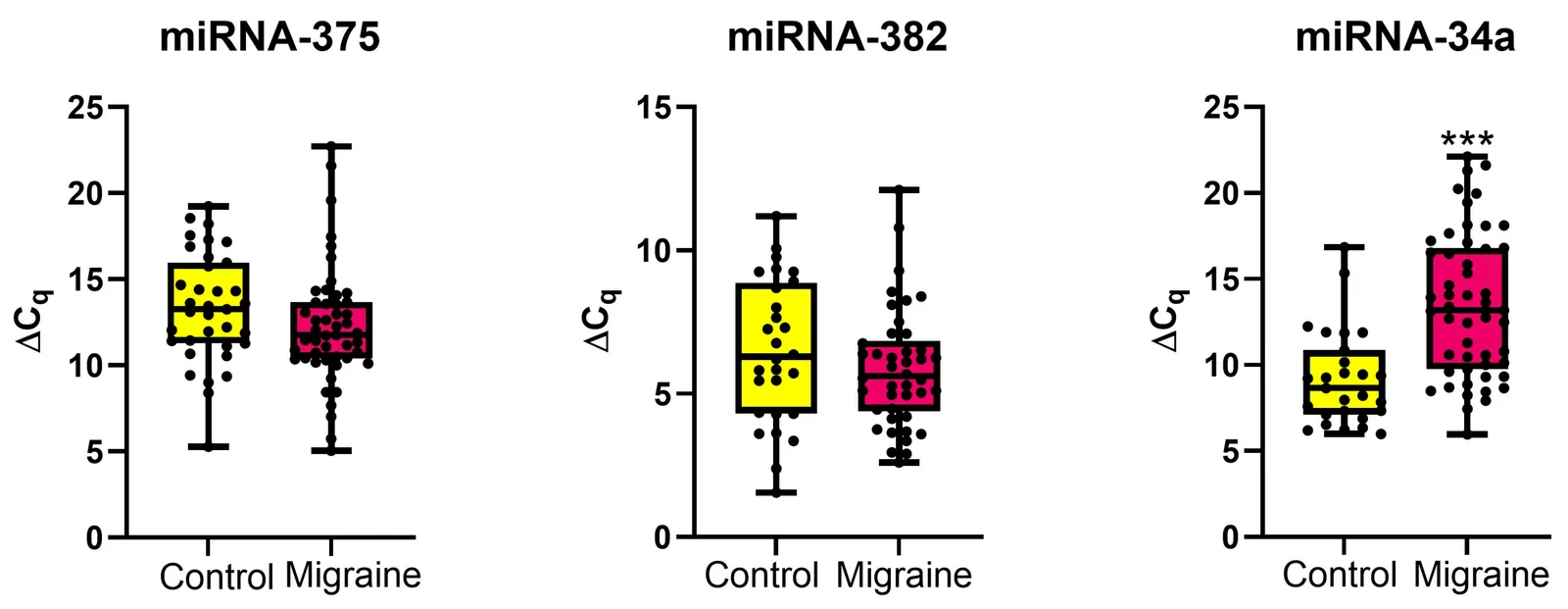
Expression levels of miRNA-375, miRNA-382 and miRNA-34a in migraine (n = 49, 42, and 51, respectively) and control (n = 35, 28, and 27, respectively). miRNA expression was assessed by quantitative real-time PCR with miRNA-24 as a reference and expressed as ΔCq values (Cq_miRNA − Cq_miRNA-24), where lower values indicate higher expression levels. Data are presented as box-and-whisker plots with individual data points. The central line represents the median, boxes indicate the interquartile range (IQR), and whiskers denote the minimum to maximum values; ***—*p* < 0.001 relative to control as evaluated by the Mann–Whitney U test.

The level of expression of mature miRNA-375, miRNA-382 and miRNA-34a is presented in Figure 3.

The expression of miRNA-34a was significantly (*p* < 0.001) lower in migraine patients than controls as indicated by higher ΔCq values. No statistically significant differences were observed between migraine and control groups for miRNA-375 and miRNA-382. The majority of values clustered within a narrow range, although a small number of higher and lower values contributed to increased variability. Overall, the distributions of expression values for miRNA-375 and miRNA-382 showed substantial overlap between groups.

### 2.5. HDAC Expression

**Figure 4:**
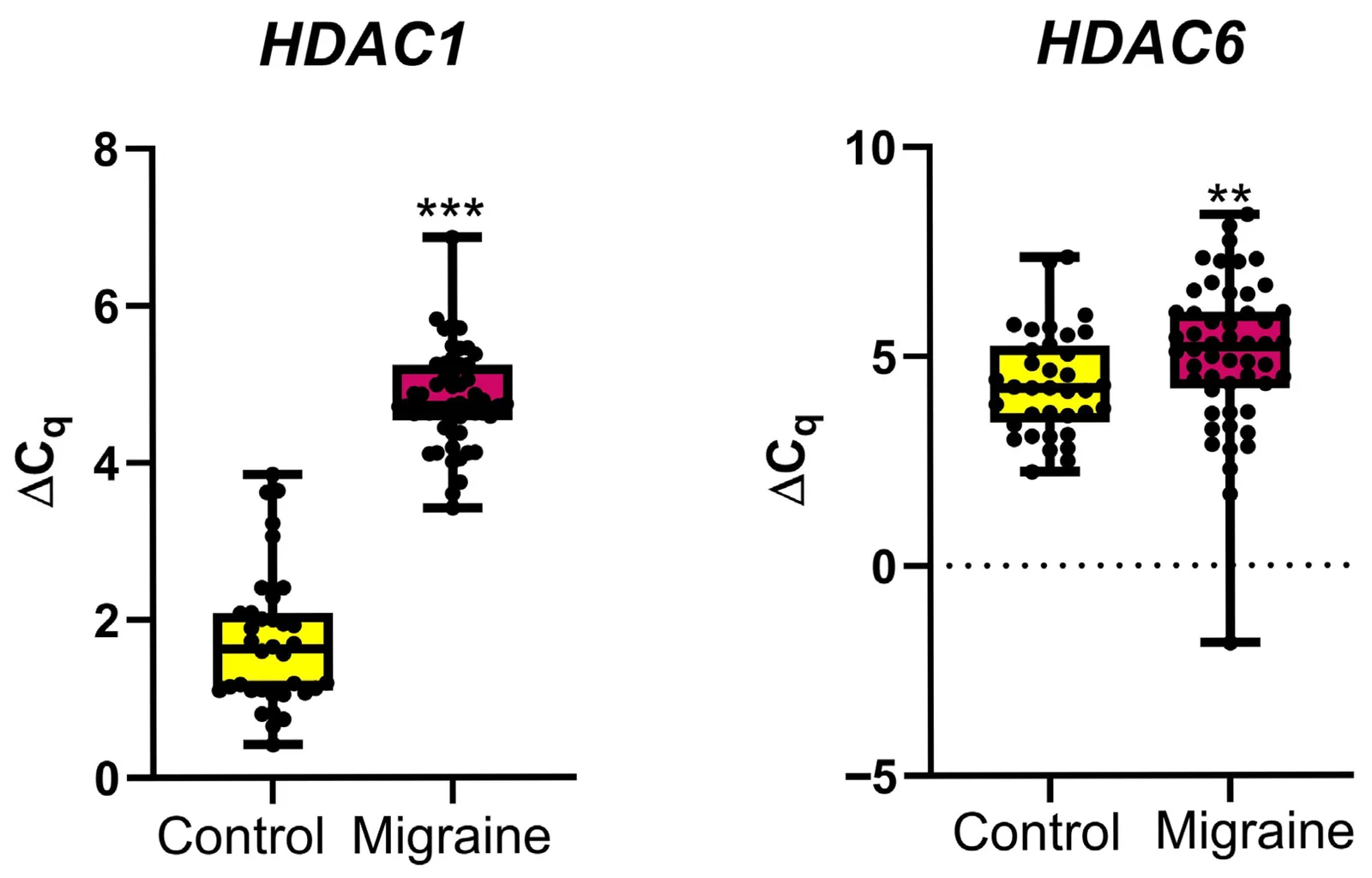
Expression levels of histone deacetylases 1 and 6 (*HDAC1* and *HDAC6*, respectively) in migraine patients (n = 54 and 52, respectively) and controls (n = 36 for both genes). *HDAC* expression was assessed by quantitative real-time PCR and expressed as ΔCq values (Cq_HDAC − Cq_reference), where lower values indicate higher expression levels. Data are presented as box-and-whisker plots with individual data points. The central line represents the median, boxes indicate the interquartile range (IQR), and whiskers denote the minimum to maximum values; **—*p* < 0.01, ***—*p* < 0.001 relative to control as evaluated by the Mann–Whitney U test.

The expression levels of the *HDAC1* and *HDAC6* genes are presented in Figure 4.

*HDAC1* expression was significantly reduced in the migraine group compared with controls, as indicated by higher ΔCq values (*p* < 0.0001). The distributions of values showed a clear separation between groups, with only a limited overlap. This represents the most prominent difference observed across all analyzed molecular parameters. *HDAC6* expression was significantly reduced in the migraine group compared with controls (*p* < 0.01). Although one extreme value was observed in the migraine group, the overall distribution of values showed a consistent shift toward higher ΔCq values.

Taken together, significant alterations were observed for miRNA-34a, *HDAC1*, and *HDAC6* expression, whereas no differences were detected in CALCA and TRPA1 expression or methylation levels.

## 3. Discussion

Searching for systemic molecular markers in migraine is important for its prevention, diagnosis, and therapy, as self-reported questionnaires, which are currently the cornerstone of migraine diagnosis, may be affected by recall bias and subjective assessment of symptoms, highlighting the need for objective, easily accessible biological markers. Consequently, peripheral blood has emerged as a valuable surrogate tissue for investigating systemic alterations that may be associated with migraine pathophysiology. Such an approach may help distinguish between local, attack-related molecular events and stable systemic alterations that could serve as clinically useful biomarkers.

The absence of differences in *CALCA* and *TRPA1* expression is particularly noteworthy given the well-established role of CGRP in migraine [6]. A double-blind crossover study demonstrated that intravenous CGRP infusion provokes migraine attacks or migraine-like headaches significantly more often than placebo in susceptible individuals [16]. Importantly, the lack of altered *CALCA* expression in peripheral blood is not inconsistent with the migraine-inducing effects of exogenous CGRP administration. While intravenous CGRP infusion clearly demonstrates functional activation of the CGRP pathway, it does not imply persistent systemic dysregulation of *CALCA* expression. Our findings suggest that CGRP-related mechanisms may operate in a transient, localized, and state-dependent manner without producing stable alterations detectable in peripheral blood during the interictal phase.

Published data concerning systemic CGRP levels in migraine are not entirely consistent. Several studies have reported increased CGRP concentrations during migraine attacks in plasma, saliva, and tear fluid, as well as during nitroglycerin-induced attacks [17,18,19,20,21,22]. Increased interictal CGRP levels have also been reported, particularly in chronic migraine patients [19,20,21,23,24]. However, other studies failed to detect elevated plasma CGRP concentrations during migraine attacks [25]. Moreover, although CGRP infusion frequently induces delayed migraine-like attacks in migraine patients, individuals without migraine typically develop only mild headaches [16,26,27,28]. Taken together, these findings indicate that activation of the CGRP pathway should not be equated with constitutive systemic dysregulation of its molecular components. Therefore, the absence of altered *CALCA* expression in the peripheral blood of episodic migraine patients during the interictal phase is compatible with the current understanding of CGRP-mediated migraine pathophysiology.

Similarly, the lack of differences in DNA methylation and in the expression of miRNA-375 and miRNA-382 further supports the notion that stable gene-specific regulatory mechanisms do not exhibit widespread alterations in peripheral blood. However, the significant reduction in miRNA-34a expression suggests that selected post-transcriptional regulatory pathways may be involved in migraine pathophysiology.

Interestingly, the observed decrease in miRNA-34a expression did not coincide with significant alterations in *CALCA* or *TRPA1* expression. This may indicate that the regulatory role of miRNA-34a extends beyond these individual targets and reflects broader changes in molecular regulatory networks associated with migraine. The results on the increased miRNA-34A expression in migraine are in line with those showing that miR-34a-5p upregulated the interleukin 1 beta (IL-1β)/cyclooxygenase 2 (COX2)/prostaglandin F2 alpha (PGE2) inflammation pathway and induced the release of CGRP via inhibition of sirtuin 1 (SIRT1) in rat trigeminal ganglion neurons [29].

In contrast, the observed downregulation of *HDAC1* and *HDAC6* suggests altered global epigenetic regulatory activity in migraine patients. Histone deacetylases play a critical role in chromatin remodeling and transcriptional regulation, and their reduced expression may indicate broader changes in epigenetic control mechanisms rather than gene-specific effects. Importantly, this finding highlights a potential dissociation between global regulatory systems and individual gene targets.

Taken together, our results suggest that migraine is not associated with stable systemic alterations in specific genes such as *CALCA* or *TRPA1*, but may involve changes at the level of epigenetic regulatory machinery. This supports a model in which migraine-related processes are dynamic and context-dependent, possibly driven by transient activation of specific pathways in relevant tissues rather than persistent systemic dysregulation.

The present study also illustrates the continued value of hypothesis-driven investigations in the era of high-throughput molecular profiling. Although genome-wide and transcriptome-wide approaches provide an unprecedented amount of information, targeted analyses remain essential for testing biologically meaningful questions derived from established disease mechanisms. In migraine, CGRP is widely recognized as a key mediator of disease pathophysiology; therefore, determining whether systemic alterations in *CALCA*- and *TRPA1*-related pathways are detectable in peripheral blood remains an important question. The lack of such alterations observed in this study is itself informative, suggesting that the biological relevance of these pathways may be restricted to specific tissues, biological compartments, or disease states rather than reflected by persistent systemic changes.

It should also be noted that expression analyses were performed using ΔCq values rather than fold-change transformation. Although fold-change values are frequently presented to facilitate biological interpretation, statistical analysis based on ΔCq values is generally considered more appropriate because ΔCq retains the original logarithmic relationship of qPCR data and avoids distortions introduced by exponential transformation. Consequently, all comparisons were performed using ΔCq values, while expression changes were interpreted accordingly. The use of ΔCq values was particularly important in the present study, as exponential transformation of qPCR data substantially increased the apparent dispersion of some measurements without altering the underlying distribution of the original data.

Several limitations of this work should be acknowledged. First, the study cohort contained a relatively high proportion of patients with migraine with aura, which may affect the generalizability of the findings. Second, the analysis was performed in peripheral blood, which may not fully reflect molecular processes occurring within the trigeminovascular system or other structures of the central nervous system directly involved in migraine pathophysiology. However, peripheral blood represents one of the few readily accessible tissues suitable for the investigation of systemic molecular alterations in living migraine patients. Third, the study focused on the interictal phase and therefore does not capture dynamic molecular changes that may occur during migraine attacks. Consequently, the present study was designed to identify stable systemic alterations rather than transient molecular changes associated with specific phases of migraine attacks. Future longitudinal studies incorporating repeated sampling during prodromal, ictal, postdromal, and interictal phases may help to further characterize dynamic regulatory mechanisms associated with migraine. Finally, despite the comprehensive approach, additional epigenetic regulatory mechanisms may also contribute to migraine pathophysiology.

In conclusion, this study provides a multi-level assessment of gene regulation in migraine and demonstrates that, despite the established role of CGRP, there is no evidence of systemic dysregulation of *CALCA* or *TRPA1* in peripheral blood. Instead, alterations in epigenetic regulatory enzymes suggest a potential shift in global regulatory mechanisms, warranting further investigation.

## 4. Patients, Materials, and Methods

### 4.1. Patients and Ethics

A total of 56 patients with migraine and 36 age- and sex-matched headache-free controls were enrolled in this study. All participants provided written informed consent prior to inclusion. Participants were recruited from the Department of Neurology and Neurology Outpatient Clinic of the Regional County Hospital in Sieradz, Poland. The study protocol was approved by the Bioethical Committee of the Polish Mother’s Memorial Hospital Research Institute, Lodz, Poland (permit no. 99/2022).

### 4.2. Diagnosis

Migraine was diagnosed according to the criteria of the International Classification of Headache Disorders, 3rd edition (ICHD-3) [4]. Only patients with episodic migraine were included in the study. Exclusion criteria for both groups comprised: organic diseases of the central or peripheral nervous system, functional neurological disorders (except for episodic migraine in the patient group), advanced or decompensated comorbidities, genetic disorders, psychiatric diseases, congenital physical or intellectual disabilities, and a history of significant multiple trauma. The control group was age- and sex-matched to migraine patients, and individuals with recurrent headaches, as well as symptoms of depression and/or anxiety (except for occasional cases with identifiable causes), were excluded.

### 4.3. Sample Collection and Processing

Peripheral blood samples were collected from all participants under standardized conditions in the morning after overnight fasting. In migraine patients, blood was collected during the interictal phase to minimize the influence of acute attack-related physiological changes on molecular analyses.

Peripheral blood mononuclear cells (PBMCs) were isolated from the buffy coat fraction. Total RNA and genomic DNA were extracted using TRIzol™ Reagent (Thermo Fisher Scientific, Waltham, MA, USA; Cat. No. 15596026) according to the manufacturer’s protocol. RNA was reverse-transcribed using the High-Capacity cDNA Reverse Transcription Kit (Thermo Fisher Scientific; Cat. No. 4368814). Genomic DNA recovered from the interphase fraction was purified, dissolved in 8 mM NaOH, and stored at −20 °C until further analysis.

The quality and concentration of nucleic acids were assessed before downstream analyses. Extracted DNA was used for DNA methylation analyses following bisulfite conversion, whereas RNA was used for gene expression studies.

### 4.4. Gene Expression

Gene expression was determined by quantitative real-time PCR (qPCR) using a Bio-Rad CFX96 C1000 Touch Real-Time System (Bio-Rad, Hercules, CA, USA). Expression of *CALCA*, *TRPA1*, *HDAC1*, and *HDAC6* was analyzed using gene-specific primers designed for this study and PerfeCTa SYBR Green FastMix ROX (Quantabio, Beverly, MA, USA). Primer sequences are provided in Supplementary Table S1. Expression levels were normalized to a reference gene and expressed as ΔCq values (ΔCq = Cqtarget − Cqreference), where lower ΔCq values correspond to higher gene expression.

### 4.5. DNA Methylation

DNA methylation of *CALCA* and *TRPA1* was assessed by methylation-specific PCR (MSP) following bisulfite conversion. Genomic DNA was bisulfite-converted using the Thermo Scientific™ EpiJET Bisulfite Conversion Kit (Thermo Fisher Scientific; Cat. No. K1461) according to the manufacturer’s instructions (Protocol A). Briefly, DNA was subjected to simultaneous denaturation and bisulfite conversion (98 °C for 10 min, followed by 60 °C for 150 min), purified, desulfonated, and eluted. Separate PCR reactions were performed for methylated (M) and unmethylated (U) DNA fractions. Primer sequences are presented in Supplementary Table S2. Relative methylation levels were expressed as ΔCq values calculated as ΔCq = CqM − CqU, where lower values indicate a higher proportion of methylated DNA.

### 4.6. miRNAs

miRNA was isolated from 300 µL of plasma using the High Pure miRNA Isolation Kit (Roche Diagnostics, Basel, Switzerland; Cat. No. 05080576001) according to the manufacturer’s instructions. Purified miRNA (350 ng) was reverse-transcribed using the TaqMan™ MicroRNA Reverse Transcription Kit (Applied Biosystems, Thermo Fisher Scientific) and miRNA-specific RT primers. Expression of mature miRNA-375, miRNA-382, miRNA-34a, and the endogenous reference miRNA-24 was determined using TaqMan™ MicroRNA Assays and TaqMan™ Universal PCR Master Mix II with UNG (Applied Biosystems, Thermo Fisher Scientific). Assay catalog numbers are presented in Supplementary Table S3. PCR amplification was performed under standard cycling conditions recommended by the manufacturer. Expression levels were normalized to miRNA-24 and expressed as ΔCq values (ΔCq = CqmiRNA − CqmiRNA-24), where lower ΔCq values correspond to higher expression levels. Technical replicates were averaged prior to analysis. Due to missing values in some measurements, samples were treated as independent observations.

### 4.7. Data Analysis

The Shapiro–Wilk W test was used to assess the normality of the data distribution for each measured parameter in the control and patient groups. For all genes, neither group followed a normal distribution, and the Mann–Whitney U test was used to assess the significance of differences between groups. Statistical power for the tests, given the sample sizes, was calculated using G*Power (Heinrich-Heine-Universität Düsseldorf, Düsseldorf, Germany, v. 3.1.9.7) [30]. For a sample size of 56 migraine patients and 36 controls, with a significance level (alpha) of 0.05, the power of our studies ranged from 0.69 to 0.81. To reduce the chance that the observed differences were due to chance, we employed the bootstrap resampling technique (10,000 iterations). Data are presented as box-and-whisker plots with individual data points. All analyses were performed with STATISTICA 13.3 software (TIBCO Software INC., Palo Alto, CA, USA) and Resampling Stats Add-In for Excel v.4 (The Institute for Statistics Education, An Elder Research Company, Arlington, VA, USA).

## 5. Conclusions

Our findings indicate that migraine is not associated with persistent systemic alterations in *CALCA* and *TRPA1* expression or DNA methylation in peripheral blood. However, significantly reduced expression of miRNA-34a, *HDAC1*, and *HDAC6* suggests the presence of alterations in broader epigenetic and post-transcriptional regulatory mechanisms. These results support a model of migraine as a dynamic disorder involving dysregulation of regulatory pathways rather than stable changes in individual target genes.

## Figures and Tables

**Table 1:** Characteristics of migraine patients enrolled in this study (*n* = 56).

Characteristic	Specification (Mean ± SD (Range) or Type and Number)
Age	41.10 ± 11.16 (18.00–71.00)
Gender	48 F ^1^/8 M
Migraine type	56 EM/0 CM
Aura	17 N/39 Y
Age at the time of the first diagnosis	28.49 ± 9.31 (14.00–50.00)
Treatment	44 A/0 P
MMD	7.48 ± 6.57 (1–44)
MHD	10.29 ± 5.81 (1.00–30.00)
MIDAS	26.37 ± 15.67 (0.00–70.00)
HIT-6	60.60 ± 8.90 (15.00–76.00)
GAD-7	9.06 ± 4.89 (1.00–21.00)
PHQ-9	7.98 ± 4.69 (0.00–19.00)

^1^ Abbreviations: F, female; M, male; EM, episodic migraine; CM, chronic migraine; A, abortive; P, preventive; MMD, monthly migraine days; MHD, monthly headache days; MIDAS, Migraine Disability Assessment Scale; HIT-6, Headache Impact Test; GAD-7, Generalized Anxiety Disorder-7; PHQ-9, Patient Health Questionnaire.

## Data Availability

The data presented in this study are available on request from the corresponding author.

## Supplementary Materials

The following supporting information can be downloaded at https://www.mdpi.com/article/10.3390/ijms27167182/s1.
