# Supplementary material for: Evidence for Altered Epigenetic Regulatory Machinery in Migraine: A Peripheral Blood Study
Source: Int J Mol Sci. 2026 Aug 11;27(16):7182. doi: 10.3390/ijms27167182 (PMC13513782; doi:10.3390/ijms27167182)
Supplement: Supplementary file 1 [file ijms-27-07182-s001.zip › ijms-4477930-supplementary.pdf]

Table S1. Sequence of the forward (F) and reverse (R) primers used for the determination of the expression of the CALCA, TRPA1, HDAC1, and HDAC6 genes and the reference gene, *RPLP0*.

Primer	Sequence (5′ → 3′)
CALCA F	CAGCTGAATGACTCAAGAAG
CALCA R	GATGCACAATAGGTAACTGC
TRPA1 F	TTTCAGGTTCCAAAGAATGC
TRPA1 R	TTGATCATTTCCAAGTCACC
HDAC1 F	CATGACTGTGTCCTTTCATAAG
HDAC1 R	GCTTGAAAATGGCCTCATAG
HDAC6 F	AATCTAGCGGAGGTAAAGAAG
HDAC6 R	TTTAACTGCTCATCCAACAC
RPLP0 F	TCTACAACCCTGAAGTGCTTGAT
RPLP0 R	CAATCTGCAGACAGACACTGG

Table S2. Sequence of the forward (F) and reverse (R) primers for methylation-specific PCR to determine DNA methylation level of the *CALA* and *TRPA1* genes.

Primer	Sequence (5′ → 3′)
CALCA M [1] F	CGTTAAATTGTTTTTAAATTTTCGT
CALCA M R	AAATCCTTTACCCCTAAATTACGTC
CALCA U F	TGTTAAATTGTTTTTAAATTTTTGT
CALCA U R	AAATCCTTTACCCCTAAATTACATC
TRPA1 M F	GCGTTGTTGTAGTTGTAGGATAGC
TRPA1 M R	TTCTAAAAAAAATTCTCAAACCCG
TRPA1 U F	TGTGTTGTTGTAGTTGTAGGATAGTG
TRPA1 U R	TTCTAAAAAAAATTCTCAAACCCAC

[1] M, target sequence DNA methylated; U, target DNA sequence unmethylated

Table S3. Catalog number of the TaqMan probe assay to determine miRNA expression.

miRNA	Catalog number	Product number
hsa-miR-24	4427975	000402
rno-miR-375*	4440886	464184_mat
hsa-miR-382	4427975	000572
hsa-miR-34a	4427975	000426
